# Two new supramolecular Ag(I) coordination polymers: luminescent properties and treatment activity on glioblastoma

**DOI:** 10.1080/15685551.2022.2041785

**Published:** 2022-02-25

**Authors:** Xiao-Feng Yan, Yu-Qiang Sun, Qing-Wei Li

**Affiliations:** a Department of Clinical Laboratory, Heilongjiang Provincial Hospital, Harbin, Heilongjiang, China; b Department of Neurosurgery, Heilongjiang Provincial Hospital, Harbin, Heilongjiang, China

**Keywords:** Ag(I) compound, mixed-ligand, luminescence, CCK-8, glioblastoma

## Abstract

Two new Ag(I) coordination polymers, namely [Ag(bpp)]·0.5 n(1,5-NDSA)·n(H_2_O) (**1**) and [Ag_2_(bpp)_2_]_n_·n(2,7-NDSA)·2 n(H_2_O)·n(CH_3_CN) (**2**) (Na_2_(1,5-NDSA) = sodium 1,5-naphthalenedisulfonate dibasic, Na_2_(2,7-NDSA) = sodium 1,5-naphthalenedisulfonate dibasic, bpp is 1,3-bis(4-pyridyl)propane), were generated *via* the solution evaporation method under room temperature. Moreover, the solids of these two compounds display strong luminescence emission at RT. And the application values of the compounds against the glioblastoma treatment were determined, and the corresponding mechanism was simultaneously tested. The analysis of CCK-8 was first implemented and the glioblastoma viability was measured. The real-time RT-PCR was next performed, and the signaling pathway activation of VEGF in glioblastoma cells was tested after treating by the above compound.

## Introduction

Gliomas are the most malignant tumors existed in central nervous system, which is one of the deadliest human malignancies. Due to their high invasiveness and easy metastasis, the survival rate of malignant tumors patients is often low, with the 2-year survival rate is less than 26.5% [1]. In accordance with the malignancy degree, gliomas are classified as grades I–IV by the World Health Organization, and the degree of malignancy is gradually increasing. Primary glioblastoma has the highest degree of malignancy, and the median progression-free survival is 6.9 months [2]. Even with standard surgical treatment, radiotherapy and adjuvant temozolomide treatment, the median overall survival is only 14.6 months [3]. Therefore, it is particularly important to find suitable therapeutic targets or effective signal pathways.

**Scheme 1.**
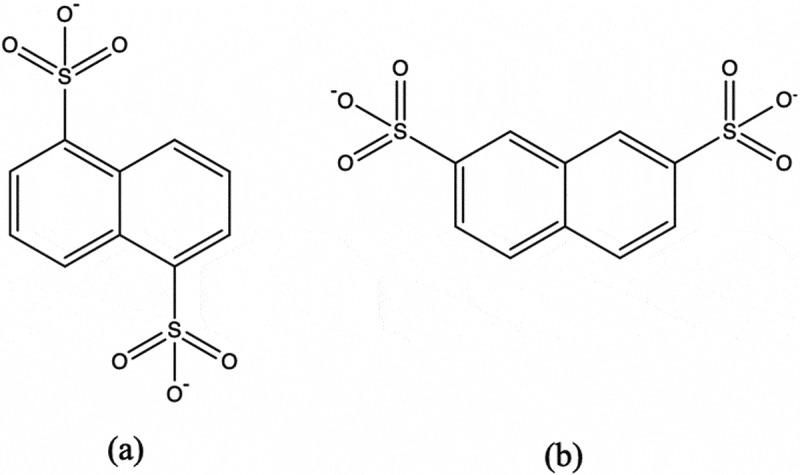
The chemical architectures of isomeric sulfonate ligands employed in the current experiment (a) 1,5-NDSA^2-^ and (b) for 2,7-NDSA^2^.

Coordination polymers (CPs) have received great interest owing to their intriguing structures and promising application potentials as functional materials used in luminescence, heterogeneous catalysis, gas storage, magnetism, nonlinear optics and so on [4–8]. For the sake of constructing desired CPs, it is critical to choose a proper organic ligand containing multiple coordination groups that have strong bond abilities with central metal ions and can bind metal ions into ordered structures. Until now, carboxylate class ligands have been widely employed to polymerize with transition metal ions for the construction of thermostable functional CPs [9–13]. Comparing to the carboxylate class ligands, sulfonate class ligands are seldom used as the organic building blocks to synthesize CPs owing to their poor coordination abilities to transition metal ions [14–16]. However, according to the reported literatures, Ag(I) ion, owing to its soft Lewis acidic property and flexible coordination environments, can be coordinated by the sulfonate ligands with the aim of N-donor ligands, extending into extended 1D, 2D or 3D frameworks [17–19]. However, recent literatures have revealed that coordination polymers have been reported to act as the role of ‘potential fluorescence content’ or ’potential anticancer reagents’. For instance, Feng and co-workers disclosed that the mixed-ligand Ag(I)-based coordination polymer showed anticancer activity toward two kinds of cancer cell lines (Hela, MCF-7) [20]; Zheng et al. reported the anticancer activity of two Co(II)-based coordination polymers toward glioma cells by inducing the apoptosis of the cells [21]. Silver complexes exhibit distinct biological activities that could be exploited to develop effective therapeutic agents including antimicrobial and anticancer drugs. The remarkable example of a silver-based drug is silver sulphadiazine that has been used topically as an antibacterial agent in the treatment of burns and wounds. Silver ions can strongly attract the sulfhydryl group of bacterial proteinase, which causes resulting in the loss of enzyme activity of the original bacteria respiration and thus the bacterial apoptosis. Ag+ is well used to prepare safe and non-carcinogenic broad-spectrum fungicides. Several studies have shown that silver coordination polymers have a wide range of anticancer activities [20,22]. For the sake of deeply extending the coordination chemistry of sulfonate class ligands, in this work, we selected two isomeric sulfonate ligands, namely 1,5-NDSA^2-^ and 2,7-NDSA^2-^ (Scheme 1), to assemble with Ag(I) ions and bpp auxiliary ligand under room temperature. Thus, we triumphantly synthesized two fresh Ag(I) CPs. X-ray diffraction analyses revealed that both compounds are based on bpp bridged 1D chain subunit. The 1,5-NDSA^2-^ and 2,7-NDSA^2-^ ligands did not coordinate directly with the Ag(I) ions. In **1**, the 1,5-NDSA^2-^ ligands form weak Ag(I) … O interactions with Ag(I) ions, which connected the bpp bridged 1D chains into a 2-dimensional layer. In the compound **2**, the ligands of 2,7-NDSA^2-^ act as hydrogen bond acceptors to form intermolecular hydrogen bonds with lattice water molecules that have weak Ag … O interactions with Ag(I) ions, which combined bpp bridged 1D chain subunits into a 3D supramolecular framework. Moreover, their luminescent performances in solid state were also explored at RT. Serial biological experiments, for instance the detection of real-time RT-PCR and CCK-8 were exploited in the current work to determine the CPs’ biological activity against the glioblastoma.

## Experimental

### Materials and instrumentation

In this work, all the chemicals for the generation of the compounds were provided by Jinan Henghua company and utilized with no further modifications. An Elementar Vario El III analyzer was performed for conducting the elemental analysis of N, H and C. TGA for the CPs were implemented with the Netzsch STA 449c thermoanalyzer with 10 °C per min raising rate under the nitrogen flow in a temperature range from 30 °C to 800 °C. With the RIGAKU DMAX2500 powder diffractometer, the characterization of PXRD could be performed with 1.54056 Å Cu/Kα radiation, at 0.05° step size. Edinburgh FLS920 spectrometer was exploited for determining luminescence emission spectra at RT. The simulated PXRD patterns were calculated using the Mercury software (Version: 4.0) [23].

### Synthesis of complexes 1 and 2

0.05 mmol of Na_2_(1,5-NDSA), 0.1 mmol of bpp and 0.1 mmol of AgNO_3_ were added into 10 mL CH_3_CN and 2 mL H_2_O, and this mixture was next stirred for half an hour. Afterwards, the mixture was filtered to remove the sediment, and the obtained filter liquor was evaporated for 3 days under room temperature to produce colorless block crystals in 32% yield in the light of AgNO_3_. Anal. Calcd. (%) for C_18_H_19_AgN_2_O_4_S (467.28): N, 5.99, C, 46.22 and H, 4.07. Found (%): N, 6.04, C, 46.19 and H, 4.05.

There’s a similarity between the synthetic process of the compound **2** and **1** but with 0.05 mmol Na_2_(1,5-NDSA) instead by 0.05 mmol Na_2_(2,7-NDSA). Colorless block crystals were produced in 28 percent of yield according to AgNO_3_. Anal. calcd. (%) for C_38_H_41_Ag_2_N_5_O_8_S_2_ (975.62): N, 7.17, C, 46.74 and H, 4.20. Found (%): N, 7.21, C, 46.72 and H, 4.17.

### X-ray crystallography

On a diffractometer of Rigaku Saturn 70 that with 0.71073 Å graphite-monochromated Mo-Kα radiation, the single crystal data of the CPs was recorded at 293 K. The direct mean together with the full-matrix least squares according to *F*^2^ were applied for the generation and modification of the **1ʹ**s structure utilizing ShelXT and OLEX2 package [24]. In the architectures, all of the non-hydrogen atoms were anisotropically modified, and all of the H atoms were fixed at the geometrical locations and isotropically modified. The CPs’ crystallographic data together with their refinements are reflected in Table 1. And their chose bond parameters are displayed in Table S1. And the H bond parameters for the CPs are reflected in Table S2.

**Table 1.** The CPs’ crystal data together with their architectural refinements

Sample	1	2
Formula	C_18_H_19_AgN_2_O_4_S	C_38_H_41_Ag_2_N_5_O_8_S_2_
*Fw*	467.28	975.62
Crystal system	monoclinic	orthorhombic
Space group	*P2_1_/n*	*Pnma*
*a* (Å)	11.487(5)	24.2583(3)
*b* (Å)	8.978(3)	17.8996(4)
*c* (Å)	18.143(6)	9.15640(10)
*α*(°)	90	90
*β*(°)	106.560(6)	90
*γ*(°)	90	90
Volume (Å^3^)	1793.5(11)	3975.84(11)
*Z*	4	4
Density (calculated)	1.731	1.630
Abs. coeff. (mm^−1^)	1.267	1.147
Total reflections	13,747	20,736
Unique reflections	4096	3882
Goodness of fit on *F^2^*	1.084	1.096
Final *R* indices [*I* > 2sigma(*I*^2^)]	*R* = 0.0457, *wR*_2_ = 0.0781	*R* = 0.0935, *wR*_2_ = 0.2328
*R* (all data)	*R* = 0.0682, *wR*_2_ = 0.0859	*R* = 0.1384, *wR*_2_ = 0.2656
CCDC	2145178	2,145,179

### CCK-8 assay

In the current paper, the detection of CCK-8 was finished to test the CPs’ inhibitory activity against the the glioblastoma viability. This research was accomplished completely abide by instructions after a few change. Shortly, in the phage of logical growth, the glioblastoma cells were gathered and inoculated into the plates (96 well) at 5000 cells per well ultimate destiny. The cells were cultivated in a 37°C incubator under 5%CO_2_. After incubation for half a day, the wells were added with compounds for finishing treatment with the sequence of concentrations. Next, the medium of cell was discarded and then the fresh medium was added involving CCK-8 reagent (10 μL). The PBS was added into the cells for treatment as control. Eventually, at 450 nm, for all wells, their absorbance was examined. This experiment was carried out for three conductions or more, and the information were displayed with mean ± SD.

### Real-time RT-PCR

The real-time RT-PCR was deeply completed for assessing the signaling pathway activation of VEGF in the glioblastoma. This present study was accomplished totally adhere to the protocols accompanied with a few change. Briefly, the glioblastoma was inoculated into the plates (6-well) at 10^5^ cells per well. The cells were cultivated in a 37°C incubator under 5%CO_2_. After incubation for half a day, the wells were added with compounds for finishing treatment with the sequence of concentrations. The PBS was added into the cells for treatment as control. The cells were next gathered and the whole RNA existed in glioblastoma cells could be extracted via exploiting TRIZOL. After testing its concentration, it was next reverse transcripted into cDNA. Eventually, in the glioblastoma, the signaling pathway activation for the VEGF was detected, utilizing *gapdh* as an internal control gene. The PBS was added into the cells for treatment as control.

## Results and discussion

### Crystal structure of compound 1

**Figure 1.**
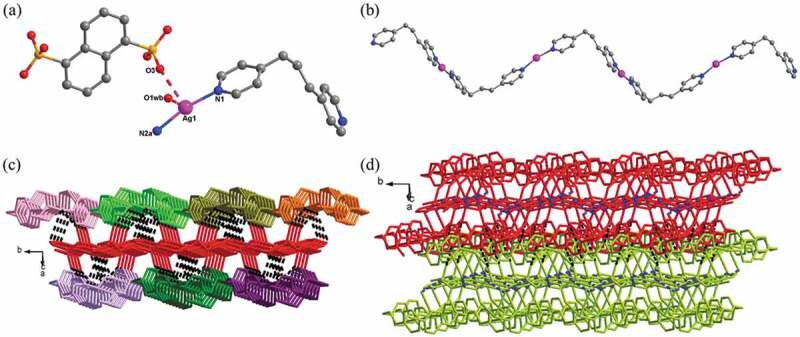
(a) The coordination surrounding diagram of Ag(I) ion in the compound **1**. (b) The 1-dimensional chain subunit consisted by bpp and Ag(I) ions. (c) The 2-dimensional layered architecture through the linkage of 1-dimensional chains by weak interactions of Ag … O (where the black dotted lines denotes weak Ag ^…^ O interactions). (d) The 3D supramolecular framework for **1** (blue dotted lines represents intramolecular hydrogen bonds in the 2D layer and the black dotted lines represent intermolecular hydrogen bonds for the construction of the 3-dimensional supramolecular structure).

The detection of single crystal X-ray diffraction displayed that the **1** belongs to the monoclinic space group of *P*2_1_/n. And its fundamental building unit is constructed from a bpp, a Ag(I) ion, a lattice H_2_O molecule, together with 0.5 1,5-NDSA^2-^ anion. As reflected in Figure 1(a), Ag(I) ion is coordinated through two N atoms originated from two bpp, employing a typical linear coordination geometry, where the lengths of Ag-N are between 2.164(3) and 2.168(3) Å, and the angle of N-Ag-N bond is 167.95°. Except for the normal coordination bonds of Ag-N, there’s also weak Ag … O interaction around Ag(I) ions, and the lengths of the Ag … O varying from 2.726 to 2.802 Å, of which the O donors come from 1,5-NDSA^2-^ anion and lattice water molecule. The bridging N-donor ligands of bpp in *cis-cis* conformation link the Ag(I) ions into a 1-dimensional infinite chain, where the distance of Ag ^…^ Ag is 12.17 Å (Figure 1(b)). Owing to the weak interactions of Ag … O, these neighboring 1-dimensional chains are connected through the 1,5-NDSA^2-^ anions to provide a 2-dimensional layer extending along plane *ac* (Figure 1(c)), which is deeply integrated through the O1w-H ^…^ O H bonds. Ultimately, the C-H ^…^ O H bonds bound the above 2-dimensional layers into a 3-dimensional supramolecular structure (Figure 1(d)). The H bond parameters for **1** have been listed in Table S2.

### Crystal structure of compound 2

**Figure 2.**
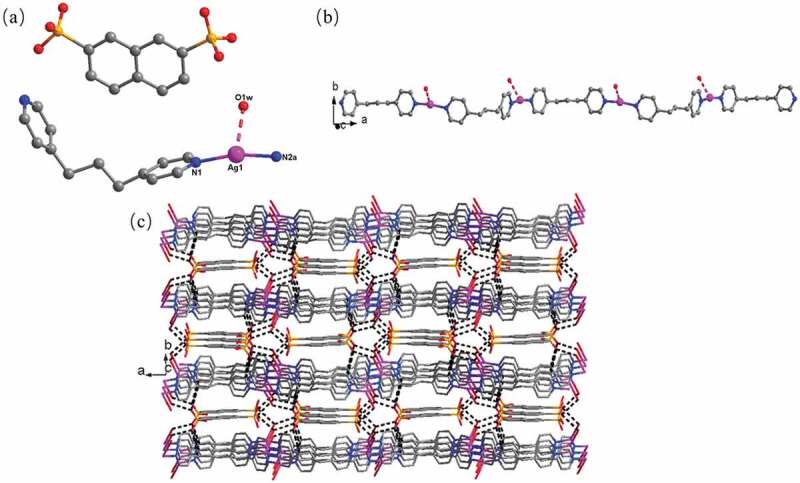
(a) The coordination surrounding diagram of Ag(I) ion in the compound **2**. (b) The 1-dimensional chain subunit consisted by bpp and Ag(I) ions. (c) The 3-dimensional supramolecular skeleton for compound **2** (where the black dotted lines denotes intermolecular H bonds).

When the 1,5-NDSA^2-^ anion was replace by 2,7-NDSA^2-^ anion, compound **2** was obtained that crystallizing in the orthorhombic crystal system with *P*nma space group. And the **2ʹ**s fundamental building unit is constituted by a bpp, a Ag(I) ion, a half 2,7-NDSA^2-^ anion, a lattice H_2_O molecule along with a lattice molecule of CH_3_CN. As displayed in Figure 2(a), the Ag(I) ion also adopts a classical linear coordination geometry that is completed through two N atoms existed in two distinct bpp, and the distance of Ag-N is 2.154(7) Å. Also, the lattice water molecule forms weak Ag ^…^ O interaction with Ag(I) ion, and length of Ag … O is 2.622 Å. Different from **1**, the free 2,7-NDSA^2-^ anion reveals no weak interaction of Ag ^…^ O with the Ag(I) ions. All of the Ag(I) ions are linked through the bpp with cis-cis conformation to generate a 1-dimensional chain (Figure 2(b)), in which the Ag ^…^ Ag distance is 12.37 Å. Because of the presence of lattice H_2_O molecules and uncoordinated sulfonic oxygen atoms, thus, suitable hydrogen bonds between finally connected these 1D chains together, resulting in the generation of a 3-dimensional supramolecular structure (Figure 2(c)). The hydrogen bond parameters for **2** have been listed in Table S2.

### Powder X-ray diffraction patterns (PXRD) and thermogravimetric analyses (TGA)

**Figure 3.**
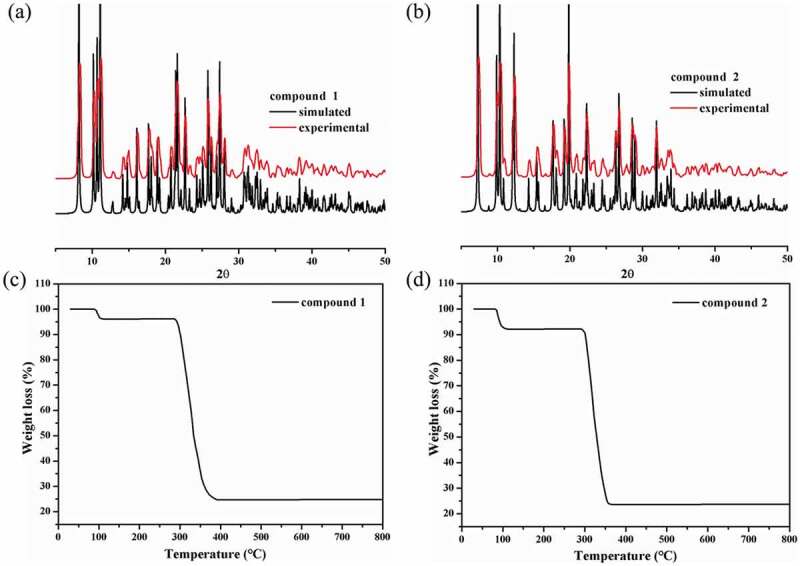
(a) and (b) The CPs’ PXRD fashions. (c) and (d) their curves of TGA.

The patterns of PXRD show that there’s no obvious difference between the experimental patterns and corresponding simulated patterns in addition to the diffraction peak intensity (Figure 3(a,b)), the phase purities of the bulk products for **1**–**2** can be demonstrated.

The stabilities of structural skeletons for **1**–**2** were also investigated *via* the thermogravimetric analysis experiments under a O_2_ atmosphere (Figure 3(c,d)). The **1ʹ**s architecture shows a two-step weight loss process: one occurred from 87 to 110 °C that is associated with the departure of lattice molecules of H_2_O (with the observed and calculated values of 3.89% and 3.85%), and the second occurred in the temperature range of 285–390 °C that was resulted from the organic ligands decomposition (with the observed and calculated values of 71.42% and 71.32%). The structure of **2** also displays a two-step process of weightlessness: the first weightlessness appearing between 80 and 112 °C was caused through the removing lattice water and CH_3_CN molecules (with the observed and calculated values of 7.84% and 7.90%), and the second occurred in the temperature range of 290–368 °C with an observed weight loss of 68.53% (calcd: 68.32%).

### Luminescent properties of compounds 1-2

**Figure 4.**
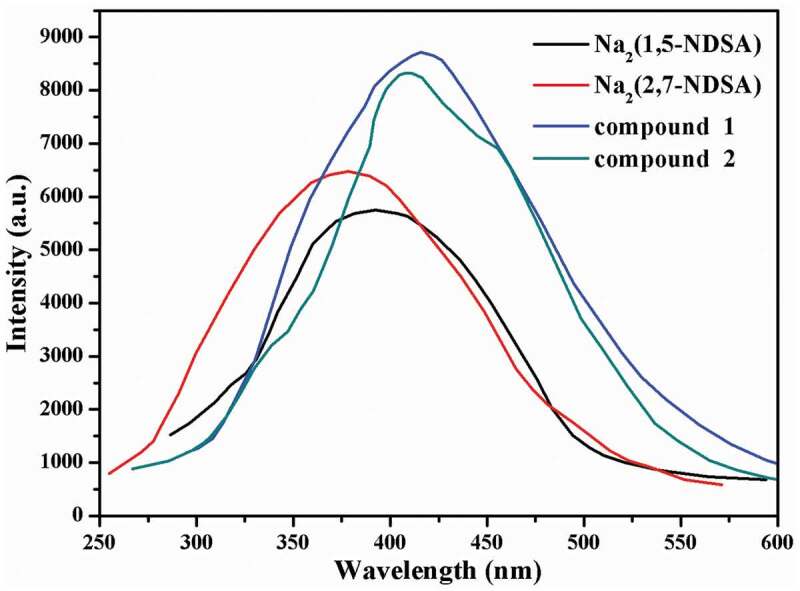
The luminescent emission spectra for compounds and sulfonate ligands.

Luminescent coordination polymers show promising applications in photochemistry and electroluminescent (EL) display [25]. Up to now, a great number of Ag(I)-based organic frameworks have been reported, and their luminescence has also been investigated [26]. In viewing of the excellent luminescent behaviors of Ag(I)-based organic coordination polymers, here we investigated the luminescent properties of compounds **1**–**2** together with their corresponding organic ligands at room temperature (Figure 4). The organic ligands of Na_2_(1,5-NDSA) and Na_2_(2,7-NDSA) used to synthesize **1** and **2** show luminescence emission, and the maximum emission peaks appearing at 392 nm and 378 nm (*λ*_ex_ = 340 nm and 340 nm), respectively, which is probably owing to the intraligand transitions of π*→*π* or π*→*π* [27]. While the ligand of bpp, as reported previously, shows no observed emission peak from 300 to 800 nm [28]. For the as-produced compounds, the emissions can be observed at approximately 415 nm and 408 nm for compounds **1** (*λ*_ex_ = 340 nm) and **2** (*λ*_ex_ = 340 nm), respectively. Notably, the CPs’ emission peaks exhibit red-shifts of 23 nm and 30 nm relative to their corresponding organic ligands. The above results indicate that the emission of compounds may originated from ligand-to-metal charge transfer [29].

### Compound significantly reduced the viability of the glioblastoma cancer cells

**Figure 5.**
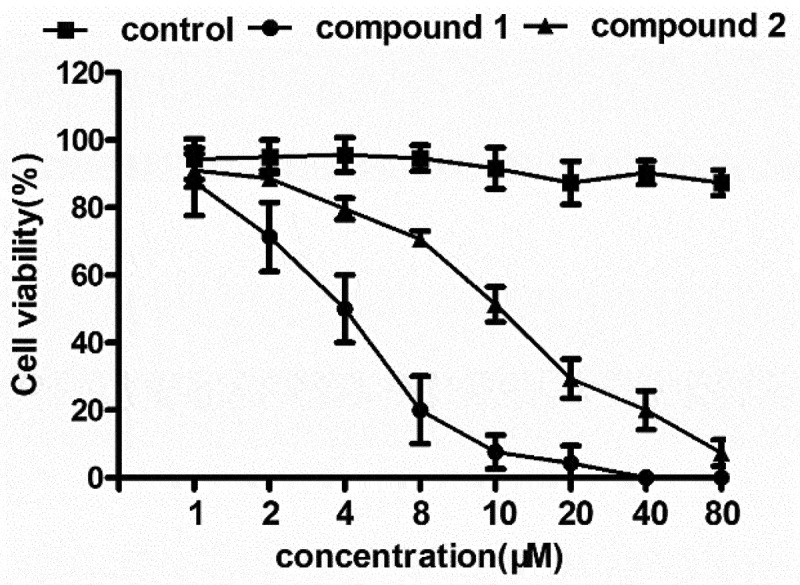
Remarkably attenuated glioblastoma viability under treating with the CPs. The complexes with various concentrations were employed to treat the glioblastoma. The glioblastoma viability could be tested with the analysis of CCK-8.

After producing the novel CPs containing fresh architectures, the application values against the treatment of glioblastoma were assessed and the corresponding mechanism was investigated simultaneously. This conduction was finished completely adhere to instructions accompanied with a few modifications. In accordance with Figure 5, in comparison with control group, the **1** displayed outstanding suppression activity against the glioblastoma viability. And there’s a markedly difference between the above groups, with P value less than 0.005. Compared with **1**, the suppression activity of the complex **2** was much weaker.

### Compound obviously inhibited the activation of the VEGF signaling pathway in the glioblastoma cancer cells

**Figure 6.**
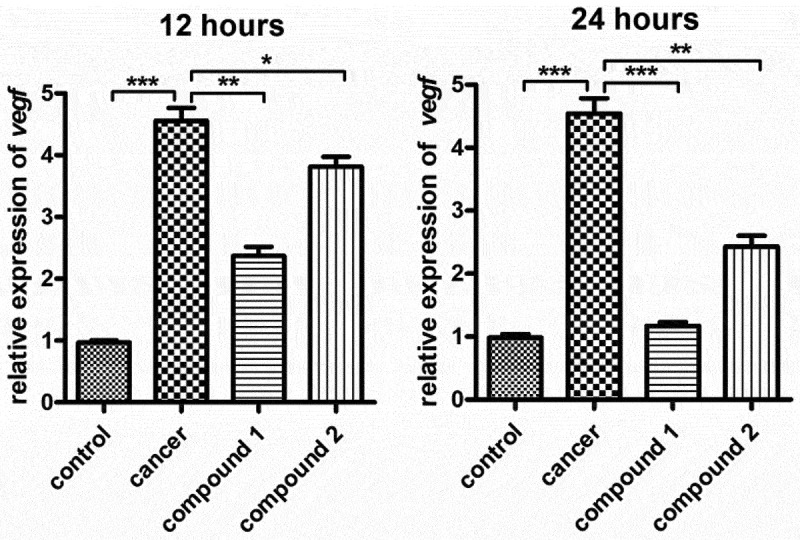
Significantly suppressed activation of signaling pathway of VEGF in glioblastoma cells under treating with the CPs. The complexes with 20 ng/ml concentrations were employed to treat the glioblastoma cells. In the glioblastoma cells, the signaling pathway activation of VEGF was also tested by real-time RT-PCR.

In the former investigation, we have verified that the **1** displayed stronger effect than **2** on attenuating the glioblastoma viability. Since signaling pathway of VEGF in glioblastoma was significant for the viability of cell. Hence, the real-time RT-PCR was deeply performed, and in the glioblastoma, the signaling pathway activation of VEGF was tested. The outcomes reflected in Figure 6 revealed that in model group, the signaling pathway activation of VEGF in glioblastoma was much superior than control group, with P value less than 0.005. After treating utilizing the **1**, in the glioblastoma cell, the signaling pathway activation of VEGF was evidently suppressed. Compared with **1**, the **2** reflected only a few effects on the signaling pathway activation of VEGF in glioblastoma cell. This outcome was in accordance with the outcomes exhibited in Figure 5.

## Conclusions

aken together, two fresh luminescent Ag(I) CPs with bpp ligands and various sulfonate ligands have been triumphantly generated and characterized structurally through utilizing single crystal X-ray diffraction. The same structural characteristics of these two compounds is the bpp ligand bridged 1D chain subunit. In **1**, the 1D chain subunits are linked *via* the weak interactions of Ag … O, which exist between Ag(I) ions and lattice 1,5-NDSA^2-^ anions, into a 2D layer, and these adjacent 2-dimensional layers are deeply bound into a 3-dimensional supramolecular structure *through* the intermolecular H bonds. In the complex **2**, the 1-dimensional chain subunits are linked together *through* the intermolecular H bonds to provide a 3-dimensional supramolecular structure. The outcomes suggest that the sulfonate ligand exert an essential effect for forming various structures. The detection of CCK-8 revealed that the **1** was much superior than **2** on suppressing the glioblastoma cells viability. Except for this, the signaling pathway activation of VEGF in glioblastoma cell was also suppressed evidently via compound **1**, which is more powerful than **2**. Ultimately, the **1** could be an outstanding candidate for treating glioblastoma through decreasing the viability of cancer cell and the signaling pathway activation of VEGF in glioblastoma. The stronger biological activity of the **1** may due to the ligand of the **1** made it enter the cell more easily than the **2**, which could be used for the further tweaking the design of the coordination polymers.

## Data Availability

Selected bond lengths (Å) and angles (^°^) for compounds **1-2** (Table S1), The hydrogen-bond parameters for compound **1** (Table S2), the information could be found in the supporting information file.
